# Tick holocyclotoxins trigger host paralysis by presynaptic inhibition

**DOI:** 10.1038/srep29446

**Published:** 2016-07-08

**Authors:** Kirat K. Chand, Kah Meng Lee, Nickolas A. Lavidis, Manuel Rodriguez-Valle, Hina Ijaz, Johannes Koehbach, Richard J. Clark, Ala Lew-Tabor, Peter G. Noakes

**Affiliations:** 1 School of Biomedical Sciences, The University of Queensland, St Lucia, 4067, Australia; 2 Queensland Alliance for Agriculture & Food Innovation, The University of Queensland, QLD, Australia; 3 Centre for Comparative Genomics, Murdoch University, Perth, WA 6150, Australia

## Abstract

Ticks are important vectors of pathogens and secreted neurotoxins with approximately 69 out of 692 tick species having the ability to induce severe toxicoses in their hosts. The Australian paralysis tick (*Ixodes holocyclus*) is known to be one of the most virulent tick species producing a flaccid paralysis and fatalities caused by a family of neurotoxins known as holocyclotoxins (HTs). The paralysis mechanism of these toxins is temperature dependent and is thought to involve inhibition of acetylcholine levels at the neuromuscular junction. However, the target and mechanism of this inhibition remain uncharacterised. Here, we report that three members of the holocyclotoxin family; HT-1 (GenBank AY766147), HT-3 (GenBank KP096303) and HT-12 (GenBank KP963967) induce muscle paralysis by inhibiting the dependence of transmitter release on extracellular calcium. Previous study was conducted using extracts from tick salivary glands, while the present study is the first to use pure toxins from *I. holocyclus*. Our findings provide greater insight into the mechanisms by which these toxins act to induce paralysis.

Ticks (order *Acarina*) are arthropods members of the class *Arachnida*, which includes spiders (order *Araneae*) and scorpions (order *Scorpionida*). These ectoparasites need to feed on the host’s blood to complete their life cycle and secrete a complex mixture of proteins in the saliva to counter host defenses^1^,^2^,^3^,^4^,^5^. A significant number of ticks, in particular from the genus *Ixodes* cause paralysis and toxicoses to the host^1^,^6^,^7^. Neurotoxins secreted in the saliva of the Australia paralysis tick (*Ixodes holocyclus*) caused an ascending flaccid paralysis and respiratory failure that can be fatal particularly in cats and dogs. The clinical symptoms of the paralysis appear in the host between three and four days after the attachment of an adult tick^8^,^9^,^10^. Other ticks that cause paralysis with major implication on human and animal health are *Dermacentor andersoni* and *Dermacentor variabilis* in North America, and *Ixodes rubicundus* and *Rhipicephalus evertis* in Africa^8^. Previous study conducted by Cooper and co-worker indicated that *I. holocyclus (Iho*) toxins inhibit the release of acetylcholine from neuromuscular junction to cause paralysis^11^. However, the target and mechanism of inhibition by these holocyclotoxins have not been determined. All former studies related with *Iho* toxins had been conducted using crude or partially purified salivary gland extract obtained from *I. holocyclus*^11^. The present study investigated the effects of the synthetic HT1, HT3 and HT12 on the skeletal neuromuscular junction using electrophysiological recordings. The current study reports that three members of the holocyclotoxin family; HT-1 (GenBank AY766147), HT-3 (GenBank KP096303) and HT-12 (GenBank KP963967) induce muscle paralysis by inhibiting the dependence of transmitter release on extracellular calcium.

## Results

**Figure 1:**
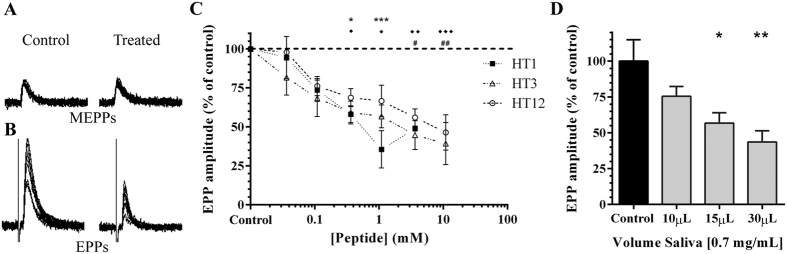
Representative recordings of (**A**), MEPP and (**B**), EPP responses during control and HT treated conditions. Sample recordings are ten consecutive responses from control and HT12 (10 mM) conditions. (**C**), dose response curves for HT1 (*n* = 4), HT3 (*n* = 3) and HT12 (*n* = 3). Normalised end-plate potential amplitudes (±SEM) were recorded at each concentration for individual HT peptides; HT1 (filled squares), HT3 (open triangles), and HT12 (open circles). Each peptide elicited a similar dose-dependent decrease in EPP amplitude, with no significant difference observed between peptides (*P* > 0.05). (**D**) dose dependent decrease in EPP amplitude in the presence of native tick saliva (0.7 mg/mL). Significance indicated is in relation to control values for respective HTs; HT1 ^*^P < 0.05 and ^***^P < 0.001, for HT3 ^♦^*P* < 0.05, ^♦♦^*P* < 0.01 and ^♦♦♦^*P* < 0.001, for HT12 ^**#**^*P* < 0.05, ^**##**^*P* < 0.01, for saliva *P < 0.05 and **P < 0.01 (One-way ANOVA with Bonferonni post test).

In this study, both native and synthesised holocyclotoxins were tested by electrophysiological analysis of neurotransmission using the mouse neuromuscular junction. End-plate potentials (EPPs) and miniature end-plate potentials (MEPPs) were recorded before, during and after HT exposure on the neuromuscular junctions. No change in the rise and decay times was noted for MEPPs at all concentrations tested for each HT peptide (Fig. 1A). MEPP frequency and amplitude were also unaffected, suggesting that the HT peptides were not targeting the calcium independent release of vesicles nor the postsynaptic acetylcholine receptors to induce paralysis. Each peptide reduced evoked EPP amplitudes in a dose dependent manner (Fig. 1B,C). No significance was found between individual HTs at each concentration tested. Incubation of preparations with native HTs resulted in a significant dose dependent decrease in mean EPP amplitude (Fig. 1D). The decrease in EPP amplitudes with maintained amplitude of MEPPs indicates the HTs act via a presynaptic mechanism. The holocyclotoxins likely change a stage between depolarisation and the calcium dependent release of vesicles, without affecting the release mechanism since MEPP frequency was unaffected.

**Figure 2:**
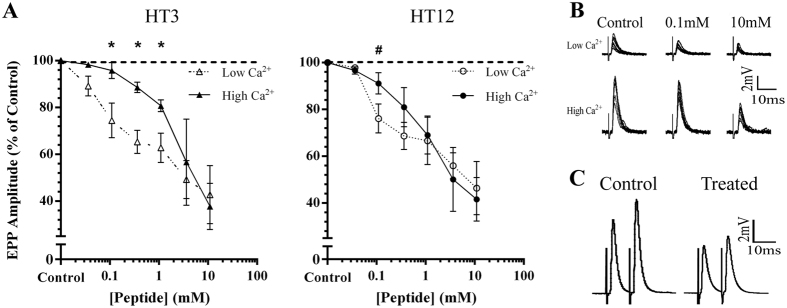
(**A**) Dose response for HT3 and HT12 conducted at low (0.3 mM) and high (2.0 mM) [Ca^2+^]. Normalised end-plate potential amplitudes (±SEM) were recorded at each concentration for HT3 at low (open triangles, *n* = 3) and high (filled triangles, *n* = 3) [Ca^2+^] and HT12 at low (open circles, *n* = 3) and high (filled circles, *n* = 4) [Ca^2+^]. At high [Ca^2+^] the effects of HT3 and HT12 were less pronounced with higher concentrations of peptide required to elicit a significant decrease in transmitter release (^*^P < 0.05, Student’s *t*-test to compare individual HT concentrations at low and high [Ca^2+^]). (**B**) representative recordings of EPP responses after incubation with HT12 at low and high [Ca^2+^]. (**C**) representative traces (mean of 10 consecutive traces) of paired pulse facilitation at 10 ms delay for control and HT12 (10 mM) treated conditions at high [Ca^2+^].

The results obtained indicate the holocyclotoxins act directly or indirectly on the VGCCs inducing a decrease in calcium entry into the terminal, which reduced the neurotransmitter release. Consistent with this mechanism was the observation that holocyclotoxins (HT 3 and HT12), were less effective in decreasing transmitter release at elevated Ca^2+^ concentration. At elevated extracellular Ca^2+^ (2.0 mM) concentrations, considerably higher concentrations of HT3 (3.0–10.0 mM) were required to significantly reduce EPP amplitude from control values (*P* < 0.001). This is in contrast to the effects of HT3 at low Ca^2+^ (0.3 mM) concentrations, under which 0.3 mM HT3 was sufficient in inducing a 35.68 ± 4.95% (*P* < 0.05) decrease in evoked amplitude. There was a significant decrease in efficacy of HT3 at 0.1, 0.3 and 1.0 mM under elevated Ca^2+^ conditions when compared with responses observed at low Ca^2+^ (*P* < 0.05) (Fig. 2A; HT3). Increased extracellular calcium also influenced the potency of HT12. EPP amplitude was decreased by 23.88 ± 6.18% (*P* < 0.05) under low Ca^2+^ (0.3 mM) conditions at concentration of 0.1 mM HT12. However, 0.1 mM HT12 reduced EPPs by 8.86 ± 2.26% (*P* > 0.05) at elevated Ca^2+^ concentrations. This effect was reversed by further increase in HT12 concentration (1–10 mM) (Fig. 2A,B). Extracellular recordings showed no effect of HTs on the nerve terminal impulse, indicating that HTs do not affect propagation of the action potential along the motor axon. These findings strongly support the presynaptic mechanism of inhibition by HT peptides, potentially via direct or indirect action on VGCCs. HTs may also indirectly act by altering the overall phosphorylation levels of presynaptic proteins involved in transmitter release. Consequently, the facilitation study in the presence of the HT12 peptide was conducted. Data showed no significant change (*P* > 0.05) in facilitation properties in the presence of HT12 (10 mM), with a facilitation index of 1.21 ± 0.06 in control recordings (*n* = 5) compared to 1.11 ± 0.08 in HT12 treated tissue (*n* = 3) (Fig. 2C).

## Discussion

Transmitter release from motor nerve terminals requires a number of prerequisites to be met: activation of P/Q-type voltage gated calcium channels (VGCCs), entry of calcium ions close to the active zone (sites of neurotransmitter release), the formation of calcium micro domains, localisation of vesicles close to the VGCCs and co-localisation of key vesicular proteins close to the vesicle and VGCCs^12^. Binding of acetylcholine to sufficient numbers of postsynaptic acetylcholine receptors is required to achieve activation of voltage gated Na^+^ channels leading to the propagation of muscle action potentials that trigger muscle contraction^13^,^14^. In the present study, intracellular and extracellular electrodes were inserted at the mouse neuromuscular junction in order to determine how these holocyclotoxins act to induce a reduction in synaptic transmission. The holocyclotoxins do not alter the amplitude; rise time or decay time of MEPPs indicating that they do not affect the acetylcholine receptors or acetylcholinesterase. However, the HTs significantly decrease the amplitude of EPPs in a dose dependent manner. Since the MEPP amplitude is unaffected, the decrease in EPPs is due to reduction in number of quanta released from the terminal which caused a decrease in quantal content. Prior study qualitatively analysed MEPPs in the presence of HTs, noting continued release even when EPP responses were blocked^11^. The findings of the present study further this observation and strongly support those of Cooper and co-worker^11^, which proposed the inhibition of transmitter release due to a reduction in quantal content, but not quantal size. Our findings suggest that HTs reduction of quantal transmitter release is mediated via a reduction in calcium entry into the nerve terminal, likely achieved by an inhibitory effect on VGCCs. It is unlikely that HTs have an effect on the vesicular associated proteins that govern the release of neurotransmitter from synaptic vesicles. On the other hand, high concentration of HTs were required to obtain a significant inhibition under our experimental conditions which can be due to the presence of unfolded toxin or difference in physiological mechanism when investigating isolated HT using this *in vitro* system. In addition, there is some evidence about possible synergetic interaction between the different holocyclotoxins (molecular weight ~5 kDa) to induce paralysis as high molecular weight holocyclotoxins from *Iho* salivary glands (40 kDa–80 kDa) have been purified and tested^15^. In this study, we have presented functional evidence indicating that members of the holocyclotoxin family inhibit neurotransmitter release via a calcium dependent mechanism resulting in a reduction of quantal content, and loss of effective neuromuscular synaptic transmission.

## Materials and Methods

### Saliva collection from fully engorged adult female *I. holocyclus*

*I. holocyclus* ticks were collected daily from cats and dogs diagnosticated with tick paralysis at veterinary clinics of the Brisbane area (Queensland, Australia). Those ticks alive that were greater in length and width than 4 mm × 3 mm respectively were salivated within 24 hours of removal from the host to ensure the toxins production. Saliva was collected following a protocol adapted from Patton and co-worker^16^. Ticks were attached to a microscope slide using sticky tape and 5 μL of 5% pilocarpine (Sigma Aldrich) in methanol was topically applied to the dorsal *scutum* of the tick, ensuring that it did not contact the basis *capitulum* and where possible, the *scutum*. The saliva was collected using 10 μL pipette tip fixed to the tick *hypostome*. Ticks were placed in an incubator at 27 °C, 75% RH. The secreted saliva was aspirated at intervals until salivation ceased. For saliva volumes greater than 2 μL, an equal volume of protease inhibitor cocktail (PIC) (Sigma Aldrich, P2714 reconstituted according to manufacturer’s instructions) was added before samples were storage at −80 °C. Saliva samples in PIC were collected throughout the 2013 tick season were pooled and the total protein concentration was measured by Bradford Assay (Bio-rad), before storage at −80 °C in aliquots.

### Synthesis of the Holocyclotoxins

Fmoc chemistry on 2-chlorotrityl resin at a 0.25 mmol scale using a CS-Bio peptide synthesizer was used for the synthesis of holocyclotoxins. Couplings were accomplished for 30 minutes with 4 eq of amino acids and 0.5 M N,N,N′,N′-tetramethyl-O-(1H-benzotriazol-1-yl)uronium hexafluorophosphate (4 eq) and *N,N*-diisopropyletylamine (4 eq) in *N,N*-dimethylformamide (DMF). Deprotection of the Fmoc reaction was conducted for 2 × 5 minutes using 20% piperidine in DMF. All the washing steps in between couplings and deprotection were in the presence of DMF. After completion of the chain assembly and final deprotection of the terminal Fmoc group peptides were cleaved off the resin using a mixture of 92% trifluoroacetic acid (TFA), 2.5% 2,2′-(ethylenedioxy)diethanethiol, 2.5% triisopropylsilane and 2.5% water (v/v). TFA was removed under reduced pressure and peptides were precipitated in ice-cold ether, resolved in 50:50 mix of buffer A (100% water, 0.05% TFA) and B (90% acetonitrile, 10% water, 0.045% TFA) (all v/v) and lyophilized. Peptides were purified by RP-HPLC on a Shimadzu Prominence HPLC unit using preparative and semipreparative Vydac C18 columns with linear gradients of 1%/min of buffer B at flow rates of 8 and 3 mL/min respectively. Oxidation was performed using either 0.1 M NH_4_HCO_3_ with 13 μM reduced glutathione (HT 1 and 2) or a mixture of 35% DMSO, 5% dodecyl-β-maltoside and 2/2 mM reduced/oxidized glutathione (HT 3) at pH 8.2 for 48–96 hours. Peptide was determined by mass spectrometry and analytical HPLC.

### Electrophysiological assay

The intracellular electrophysiological recordings were conducted using the *extensor digitorum longus* (EDL) muscles from 30-day-old C57BL/6-129SvJ mice. All animal experimental procedures were approved by The University of Queensland Animal Care and Ethics Committee (Ethics number 152/12) and complied with the Australian Code of Practice for the Care and Use of Animals for Scientific Purposes. Nerve-muscle preparations and recording procedures were conducted as per our previous work^17^ and all electrophysiology experiments were conducted in triplicate. In short, extracted EDL muscle with intact innervating motor nerve were dissected free and pinned to a 5 mL recording chamber. Preparations were perfused with standard Tyrode’s solution (1.0 mM [Mg^2+^]) and continuously gassed with 95% O_2_ and 5% CO_2_ at room temperature (23 ± 2 °C). The bath calcium was 0.3 mM for characterisation of HTs and increased to 2.0 mM to investigate changes at elevated [Ca^2+^]. The innervating motor nerve was supra-maximally stimulated directly by square wave pulses of 0.10 ms duration and 10–20 V amplitude. Stimulus-induced muscle contractions were prevented by the use of sodium channel blocker, μ-Conotoxin GIIIB (0.1–0.5 μM, Alomone Labs). Fine tipped glass microelectrodes (30–50 MΩ) filled with 2 M KCl EPPs and MEPPs. Once a stable site was found and control recordings taken, the stimulus was halted to allow synaptic vesicle replenishment. Control recordings were taken in standard Tyrode’s solution described above in the absence of HTs. HT peptides, diluted in Tyrode’s solution (1 mg/mL) and tick saliva, were applied directly to the tissue and left to incubate for a thirty-minute period, after which the stimulus commenced and intracellular recordings were taken. This process was repeated for each HT concentration and saliva trialled.

## Additional Information

**How to cite this article**: Chand, K. K. *et al*. Tick holocyclotoxins trigger host paralysis by presynaptic inhibition. *Sci. Rep.* **6**, 29446; doi: 10.1038/srep29446 (2016).

## References

[b1] Jongejan F. & Uilenberg G. The global importance of ticks. Parasitology 129, S3–S14, doi: 10.1017/S0031182004005967 (2004). 15938502

[b2] Rodriguez-Valle M. . Comparative microarray analysis of *Rhipicephalus (Boophilus) microplus* expression profiles of larvae pre-attachment and feeding adult female stages on *Bos indicus* and *Bos taurus* cattle. BMC Genomics 11, 47, doi: 10.1186/1471-2164-11-437 (2010). 20637126 PMC3224725

[b3] Ribeiro J. M., Anderson J. M., Manoukis N. C., Meng Z. & Francischetti I. M. A further insight into the sialome of the tropical bont tick, *Amblyomma variegatum*. BMC Genomics 12, 136, doi: 10.1186/1471-2164-12-136 (2011). 21362191 PMC3060141

[b4] Francischetti I. M., Sa-Nunes A., Mans B. J., Santos I. M. & Ribeiro J. M. The role of saliva in tick feeding. Frontiers in bioscience 14, 2051–2088 (2010). 10.2741/3363 PMC2785505 19273185

[b5] Chmelar J., Kotál J., Kopecky J., Pedra J. H. F. & Kotsyfakis M. All For One and One For All on the Tick–Host Battlefield. Trend in Parasitology 1476, doi: http://dx.doi.org/10.1016/j.pt.2016.01.004 (2016). 10.1016/j.pt.2016.01.004 PMC4851932 26830726

[b6] Mans B. J., Gothe R. & Neitz A. W. Biochemical perspectives on paralysis and other forms of toxicoses caused by ticks. Parasitology 129 Suppl, S95–111 (2004). 15938507 10.1017/s0031182003004670

[b7] Guglielmone A. A., Estrada-Pena A., Luciani C. A., Mangold A. J. & Keirans J. E. Hosts and distribution of *Amblyomma auricularium* (Conil 1878) and *Amblyomma pseudoconcolor Aragao*, 1908 (Acari: *Ixodidae*). Exp Appl Acarol 29, 131–139 (2003). 14580065 10.1023/a:1024251020035

[b8] Masina S. & Broady K. W. Tick paralysis: development of a vaccine. International Journal for Parasitology 29, 535–541 (1999). 10428629 10.1016/s0020-7519(99)00006-5

[b9] Eppleston K. R., Kelman M. & Ward M. P. Distribution, seasonality and risk factors for tick paralysis in Australian dogs and cats. Veterinary Parasitology 196, 460–468, doi: 10.1016/j.vetpar.2013.04.011 (2013). 23643358

[b10] Thurn M. J., Gooley A. & Broady K. W. In Recent Advances in Toxinology Research Vol. 2 (ed Gopalakrishnakone P., Tan C. K. Ž.) 243–256 (Venom and Toxin Research Group, National University of Singapore, 1992).

[b11] Cooper B. J. & Spence I. Temperature-dependent inhibition of evoked acetylcholine release in tick paralysis. Nature 263, 693–695 (1976). 185525 10.1038/263693a0

[b12] Urbano F. J. . Altered properties of quantal neurotransmitter release at endplates of mice lacking P/Q-type Ca^2+^ channels. Proc Natl Acad Sci USA 100, 3491–3496, doi: 10.1073/pnas.0437991100 (2003). 12624181 PMC152320

[b13] Flucher B. E. & Daniels M. P. Distribution of Na^+^ channels and ankyrin in neuromuscular junctions is complementary to that of acetylcholine receptors and the 43 kDa protein. Neuron 3, 163–175 (1989). 2560390 10.1016/0896-6273(89)90029-9

[b14] Wood S. J. & Slater C. R. Beta-Spectrin is colocalized with both voltage-gated sodium channels and ankyrinG at the adult rat neuromuscular junction. J. Cell Biol. 140, 675–684 (1998). 9456326 10.1083/jcb.140.3.675 PMC2140176

[b15] Stone B. F. Chemical characterization studies on salivary gland toxins of the paralysis tick *Ixodes holocyclus*, in Neurotoxins: Fundamental and Clinical Advances (Edrs. Chubb I. W. & Geffen L. B.), 273 (Adelaide University, Union Press, Adelaide, 1979).

[b16] Patton T. G., Dietrich G., Brandt K., Dolan M. C., Piesman J. & Gilmore R. D. Jr. Saliva, salivary gland, and hemolymph collection from *Ixodes scapularis* ticks. J. Vis. Exp. e3894 (2012). 10.3791/3894 PMC3912584 22371172

[b17] Chand K. K., Lee K. M., Schenning M. P., Lavidis N. A. & Noakes P. G. Loss of beta2-laminin alters calcium sensitivity and voltage-gated calcium channel maturation of neurotransmission at the neuromuscular junction. J Physiol 593, 245–265, doi: 10.1113/jphysiol.2014.284133 (2015). 25556799 PMC4293066

