# Author Correction: Tick holocyclotoxins trigger host paralysis by presynaptic inhibition

**DOI:** 10.1038/s41598-022-24528-4

**Published:** 2022-11-30

**Authors:** Kirat K. Chand, Kah Meng Lee, Nickolas A. Lavidis, Manuel Rodriguez-Valle, Hina Ijaz, Johannes Koehbach, Richard J. Clark, Ala Lew-Tabor, Peter G. Noakes

**Affiliations:** 1 School of Biomedical Sciences, The University of Queensland, St Lucia, 4067 Australia; 2 Queensland Alliance for Agriculture & Food Innovation, The University of Queensland, St Lucia, QLD Australia; 3 Centre for Comparative Genomics, Murdoch University, Perth, WA 6150 Australia

Correction to: *Scientific Reports* 10.1038/srep29446, published online 08 July 2016

This Article contains errors.

Some of the descriptions of the methods have been reused from a source which was not cited. The citation should have appeared in text as follows and is referenced in this notice as Reference 1:

## Materials and methods

### Saliva collection from fully engorged adult female *I. holocyclus*

*I. holocyclus* ticks were collected daily from cats and dogs diagnosed with tick paralysis at veterinary clinics of the Brisbane area (Queensland, Australia) as recently described^1^. Those ticks alive that were greater in length and width than 4 mm × 3 mm respectively were salivated within 24 hours of removal from the host to ensure the toxins production. Saliva was collected following a protocol adapted from Patton and co-worker^16^. Ticks were attached to a microscope slide using sticky tape and 5 μL of 5% pilocarpine (Sigma Aldrich) in methanol was topically applied to the dorsal scutum of the tick, ensuring that it did not contact the basis capitulum and where possible, the scutum. The saliva was collected using 10 μL pipette tip fixed to the tick hypostome. Ticks were placed in an incubator at 27 °C, 75% RH. The secreted saliva was aspirated at intervals until salivation ceased. For saliva volumes greater than 2 μL, an equal volume of protease inhibitor cocktail (PIC) (Sigma Aldrich, P2714 reconstituted according to manufacturer’s instructions) was added before samples were storage at − 80 °C. Saliva samples in PIC collected throughout the 2013 tick season were pooled and the total protein concentration was measured by Bradford Assay (Bio-rad), before storage at − 80 °C in aliquots^1^.

Additionally, the Acknowledgement section in the Article is incomplete. The correct statement should read:

#### **Acknowledgements**

This project was supported by The University of Queensland/Australian Research Council linker project -LP120200836, and KKC was supported by Australian postgraduate awards (APA) scholarship. ARC Future Fellowship (FT100100476) supported RJC and JK by University of Queensland Postdoctoral Fellowship. The authors wish to acknowledge that the *Ixodes holocyclus* tick saliva used in this study was collected and provided by Ms Greta Busch, Centre for Animal Science, QAAFI, The University of Queensland.
